# Experimental Study for the Stripping of PTFE Coatings on Al-Mg Substrates Using Dry Abrasive Materials

**DOI:** 10.3390/ma13030799

**Published:** 2020-02-10

**Authors:** Guillermo Guerrero-Vaca, David Carrizo-Tejero, Óscar Rodríguez-Alabanda, Pablo E. Romero, Esther Molero

**Affiliations:** Department of Mechanical Engineering, University of Cordoba, Medina Azahara Avenue, 5, 14071 Cordoba, Spain; guillermo.guerrero@uco.es (G.G.-V.); davidc179@hotmail.com (D.C.-T.); p62rocap@uco.es (P.E.R.); esther.molero@uco.es (E.M.)

**Keywords:** stripping, abrasives, PTFE coating, fluoropolymer coating, corundum, glass microspheres, plastic particles, walnut shell

## Abstract

Polytetrafluoroethylene (PTFE) coatings are used in many applications and processing industries. With their use, they wear out and lose properties and must be replaced by new ones if the cost of the element so advises. There are different stripping techniques, but almost all of them are very difficult and require strict environmental controls. It is a challenge to approach the process through efficient and more sustainable techniques. In the present work, we have studied the stripping of PTFE coatings by projection with abrasives (1 step) as an alternative to carbonization + sandblasting procedures (2 steps). For this purpose, different types of abrasives have been selected: brown corundum, white corundum, glass microspheres, plastic particles, and a walnut shell. The tests were performed at pressures from 0.4 to 0.6 MPa on PTFE-coated aluminium substrates of EN AW-5182 H111 alloy. Stripping rates, surface roughness, and substrate hardness have been studied. Scanning electron microscopy (SEM) images of sandblasted specimens have also been obtained. All abrasives improved mechanical and surface properties in one-step vs. two-step processes. The abrasives of plastic and glass microspheres are the most appropriate for the one-step process, which increases the hardness and roughness level Ra in the substrate. Corundum abrasives enable the highest stripping rates.

## 1. Introduction

Many industries use surface coatings for special applications [1]. Techniques for surface coating are used in the automotive industry [2], in the metal industry, in the aviation sector [3], in the food processing industries [4], in the biomedical sector [5], and in the chemical industry [6], among many others.

Coatings can be metallic, polymeric, ceramic, and organic. Application methods include electrodeposition, plasma spraying, hot dipping, chemical conversion coatings, physical and chemical vapour deposition, thermal spraying, paints, glass enamels, and sol-gel deposition.

This work is done with polymeric coatings obtained by applying fluorinated paints on elements used in the food sector.

In the breadmaking industry and related products, in the plastic containers and packaging manufacturing sector, in the chemical industry, or in the meat industry among others, part of the metallic elements (tools, moulds, trays, and reactors) are protected with anti-adherent fluoropolymer coatings [7]. The objective is multiple: to prevent adherence to substrates, to give low chemical reactivity, to facilitate cleaning, to improve unmoulding, and to increase the life of the elements [8]. The fluoropolymer-based resins usually used for these coatings are [9]: polytetrafluoroethylene (PTFE), perfluoroalkoxide (PFA), and fluorinated ethylene propylene (FEP). However, due to lower surface energy and very low chemical reactivity, the most common is PTFE [10,11]. Once coated, the metal elements must pass through a furnace at about 400–420 °C for sintering the coating.

Coatings have a limited service life. After a certain number of uses, they must be removed and the metallic elements must be protected again [12]. There are several techniques to remove the coating: high pressure water jet, chemical procedures, carbonization, plasma, cryogenic techniques, high intensity light pulses, laser, and more.

Considering the qualities of low chemical reactivity, high surface hardness, high adhesion capacity on the substrate, and the ability to remain unchanged until relatively high temperatures (500 °C), PTFE coatings are very difficult to remove [13]. Therefore, most of the techniques previously listed produce inefficient results [14] or, as the case may be, require high operational and environmental control costs, which makes it advisable to discourage their use [15].

Sandblasting is another common coating removal technique. In the literature, works related to abrasive stripping applications in different sectors can be found such as civil steel structures [16], castings [17], paint stripping on aircrafts [18], paint stripping on ships [19], and for removing graffiti [20].

For the removal of non-stick fluoropolymer coatings, it is common to perform a two-step process. In the first step, a carbonization is performed [15,21]. In the second step, the carbonized coating is removed by sandblasting with corundum-type abrasives [21]. The carbonisation stage requires rigorous and high costs to ensure adequate environmental control to minimise risks for operators. It is challenging to efficiently resolve the removal of these coatings in only one step and without carbonization. The development of new types of abrasives [22], the increasingly widespread introduction of automated systems [23,24], and the existence of stricter regulatory standards [25] have all led to a renewed commitment to abrasive projection stripping procedures [26]. This study evaluated the use of several abrasives with a wide range of characteristics, including white corundum, brown corundum, glass microspheres, plastic particles, and a walnut shell [27].

In the food processing industry, in the metal forming industry, and in the chemical industry, light alloys are used as a substrate. One family of materials commonly used are aluminium alloys. Among them, the 5000 series (aluminium-magnesium) is of particular interest. These alloys have several advantages. They allow food contact with certain limitations [28] while being suitable for cold forming. Furthermore, they have adequate weldability and excellent corrosion resistance. In our study, the EN AW 5182 alloy with PTFE coatings was used.

The objective of this work is to determine and compare the efficiency of the use of various abrasives in the stripping of PTFE coatings on Al-Mg alloys in a single step, with the aim of analysing the impact on the mechanical and surface properties on the substrate to propose the optimal abrasives.

## 2. Materials and Methods

For this work, 45 square specimens of 25 mm × 25 mm × 5 mm of magnesium aluminium alloy EN AW 5182 H111 supplied by Broncesval (Paterna, Valencia, Spain) have been used. These test pieces are necessary to carry out 15 tests with five abrasives at three pressures. The tests have been repeated three times.

The results obtained for the chemical composition by X-ray microanalysis (EDX) with a JEOL JSM 6300 (Jeol USA, Peabody, MA, USA) are shown in Table 1 and are compatible with the standard compositions.

This aluminium alloy, in the state of supply, is an annealed alloy in which its final acridity degree has been obtained by cold rolling. The Vickers hardness in this state is 85.0 HV5 and it has been measured with a Zwick/Roell ZHU250 (Zwick Iberica Testing Equipment S.L, San Cugat del Valles, Barcelona, Spain), according to UNE-ISO 6507 [29].

The coating is called as TFI-2531 N by Tecnimacor (Tecnimacor S.L, Córdoba, Spain) who is a specialist in the application of fluoropolymeric non-stick coatings. TF-2531 is a fluoropolymer-based on PTFE. It is a three-layer coating. The applied products have been supplied by the Whitford Company (Whitford España S.L, Barberá del Vallés, Barcelona, Spain). The first layer is a liquid resin applied by spraying with an HVLP gun (high volume low pressure). After the drying of the first layer, a second layer is applied and then a third layer, wet-on-wet. Lastly, the whole is cured in an NA 15/65 electric oven (Nabertehem GmbH, Lilienthal, Germany). The characteristics of the coating are shown in Table 2.

**Figure 1:**
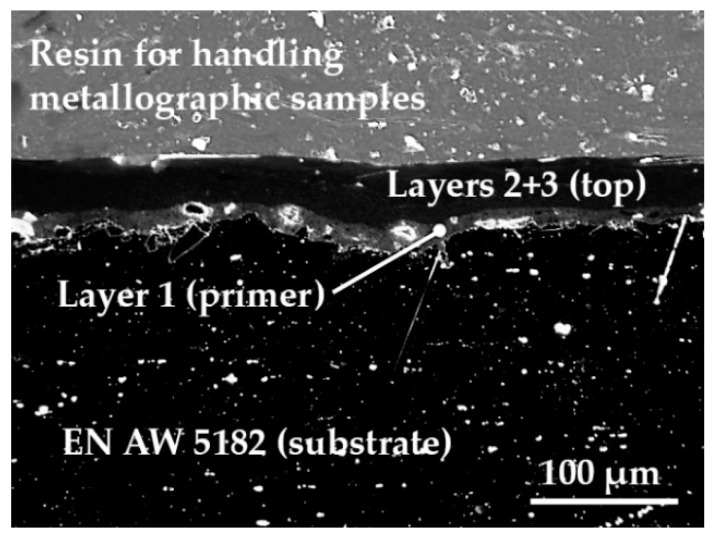
EN AW-5182 Al-Mg alloy substrate with PTFE coating.

A micrography showing the cross-section of the aluminium substrate with the applied coating is provided in Figure 1 to show the morphology of the deposited layers.

The structure of the Al-Mg alloy is recrystallized after the application of the curing heat treatment (410 °C for 15 min) on the PTFE coating, which shows a decrease in hardness with respect to the state of the supply (from 85.0 to 71.3 HV5).

Five types of abrasives, with the properties and characteristics indicated in Table 3, were used for the tests. Other abrasives such as silica have been discarded due to the limitations of their use in closed spaces, or metallic abrasives that have a high risk of becoming embedded in the surface of the aluminium and produces oxidation. Other abrasives such as sodium bicarbonate are commonly used in wet media and this study has been focused on dry media.

**Figure 2:**
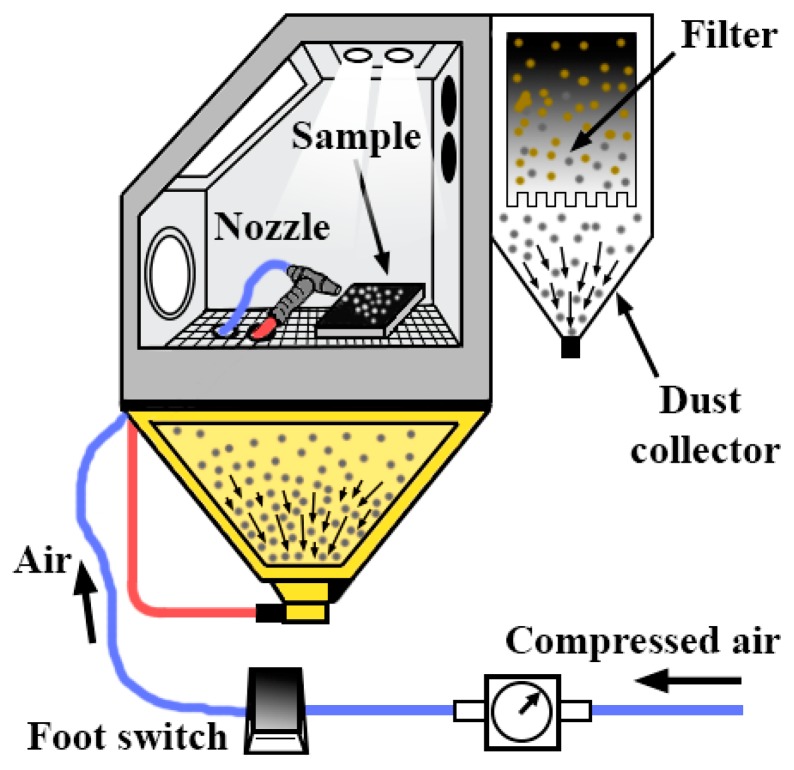
Diagram and assembly of abrasive particle spraying equipment.

Suction equipment (venturi effect) was used for the projection of the abrasive particles. It is about the model Sand Blast Cabinet CAT-990 (Aslak S.L, San Quirze del Valles, Barcelona, Spain). The projection nozzle has a diameter of 6.5 mm. The projection was made at a distance of 200 mm from the substrates and at 90° with a pressure of 0.4, 0.5, and 0.6 MPa. Figure 2 shows the assembly.

The roughness of the substrate after stripping has been measured by a Mitutoyo SJ-201 roughness tester (Mitutoyo Corporation, Sakada, Japan). Ra and Rz have been measured.

The coating-stripped surfaces were checked by visual inspection. The coating is black and the removal contrasts sharply with the colour of the aluminium.

The SEM images were obtained with the high-resolution scanning electron microscope FE-SEM JEOL JSM 7800F Prime (JEOL USA, Inc., Peabody, MA, USA).

## 3. Results and Discussion

### 3.1. Stripping Rate

**Figure 3:**
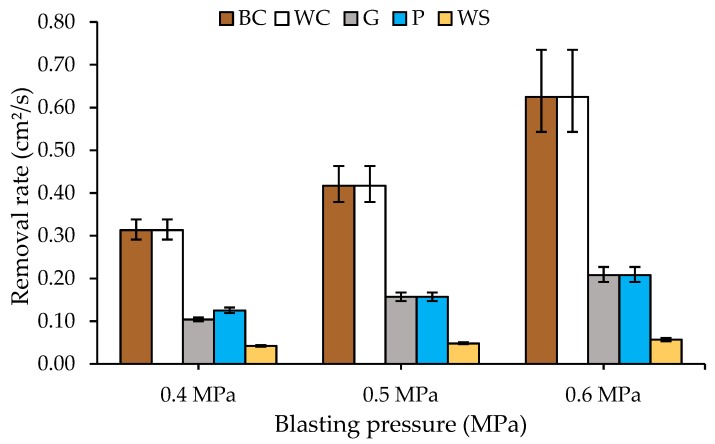
Stripping rate on PTFE coatings on EN AW-5182 substrate vs. spray pressure with different abrasives: (i) BC-brown corundum, (ii) WC-white corundum, (iii) G-glass microspheres, (iv) P-plastic particles, and (v) WS-walnut shell.

The stripping rate has been obtained once the surface is free of PTFE coating. Figure 3 shows the stripping rates in cm^2^/s, depending on the abrasive material and the pressure used.

It is evident that, for both brown corundum (BC) and white corundum (WC), the stripping rates are similar, which is the highest in the experiments. They vary between 0.3 and 0.6 cm^2^/s. For glass microspheres (G) and plastic particles (P), intermediate stripping rates (between 0.1 and 0.2 cm^2^/s) were obtained. For the abrasive walnut shell (WS), the stripping rate is barely 0.1 cm^2^/s. The highest stripping rates were obtained at high pressures and with the hardest abrasive materials. This result is consistent with that obtained for plastic particles in metals [30], for glass microspheres on stainless steel 316 L [31], and for titanium surfaces in the case of corundum [32].

The best option to maximize the stripping rate in the proposed system are both white and brown corundum abrasives, applied at 0.6 MPa projection pressure showing stripping rate values between 0.6 and 0.7 cm^2^/s. These values are lower than those obtained by a two-step process (carbonization + sandblasting) in which rates of up to 1 to 1.2 cm^2^/s are reached [33].

### 3.2. Surface Roughness

**Figure 4:**
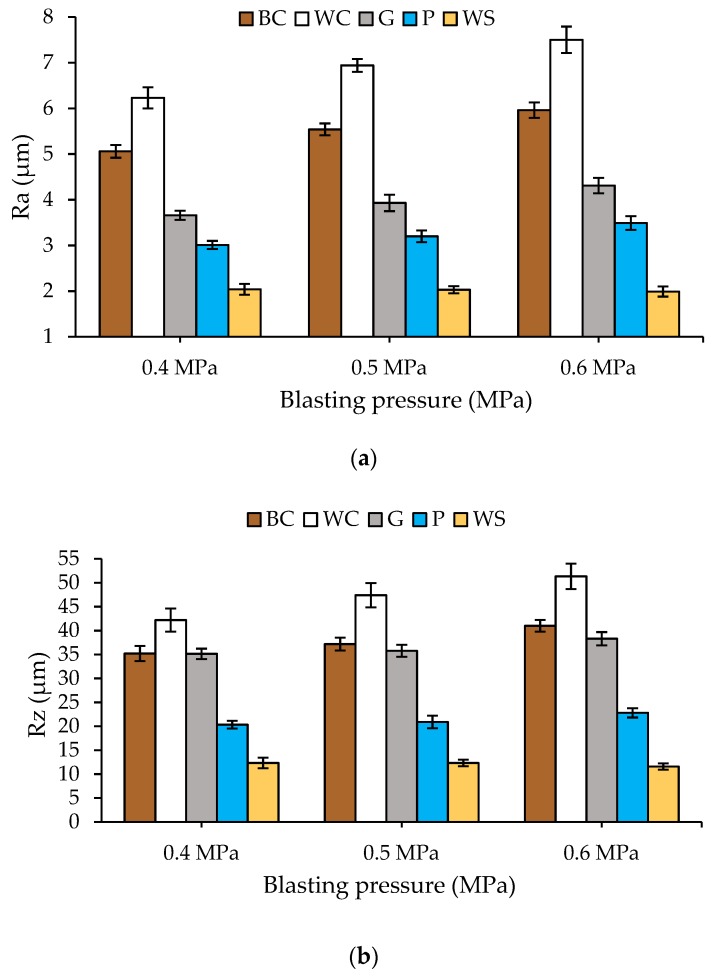
Ra (**a**) and Rz (**b**) values after stripping PTFE coatings on the EN AW 5182 substrates with different abrasives: (i) BC-brown corundum, (ii) WC-white corundum, (iii) G-glass microspheres, (iv) P-plastic particles, and (v) WS-walnut Shell.

Surface roughness is a relevant aspect when reusing substrates for a new application of PTFE coatings. The greater or lesser ease with which the new coatings remain attached to the surface of the reused aluminium will largely depend on the roughness values of the substrate. The literature recommends values of Ra between 2.5 to 4 µm [7,10,12]. The roughness values obtained in the experiments performed in this work are shown in Figure 4.

The results showed that the highest values were obtained for white corundum, which was followed by brown corundum. As in the case of stripping rates, the relations between hardness of the abrasive and pressure used are the same. The higher the hardness and the projection pressure of the abrasive are, the higher the roughness value is. It is worth mentioning the low roughness generated by the abrasive walnut shell.

These results are consistent with the shape, hardness, and projection pressure of the abrasives [34]. The roughness values (Ra) closest to target 2.5–4 μm are produced by the plastic particles at all pressures tested and the glass microsphere for pressures between 0.4–0.5 MPa. In the two-step process, the values obtained are significantly lower and always between 1.5–2 µm of Ra [21].

The geometry and hardness of the abrasives produce textures that mark the surface of the stripped substrate to a certain depth and are related to the angle of impact, in our case 90°, and the hardness difference between the abrasive and the substrate. The depth of these marks, their distribution, and shape are consistent with the type of abrasive used in other works [34,35,36].

### 3.3. Hardness of Substrates

Aluminium-magnesium substrates coated with PTFE suffer high thermal stress and their structure is recrystallized after the coating has cured. This recrystallized structure reduces hardness and mechanical properties [33]. It is, therefore, interesting to increase the hardness of the substrate to solve, at least partially, the loss of properties provoked by heat treatment. It is important to note the depth to which the hardening has penetrated into the total thickness of the substrate. The projection of particles produces a hardening [37] since the particles’ impact on the substrate and the density of defects (dislocations) in the crystalline structure increase, which hardens it due to this mechanism. Similarly, mechanical fatigue behaviour can be improved [38]. This last aspect is not dealt with in this work because most of the applications of these coated parts are for substrates with static charges.

**Figure 5:**
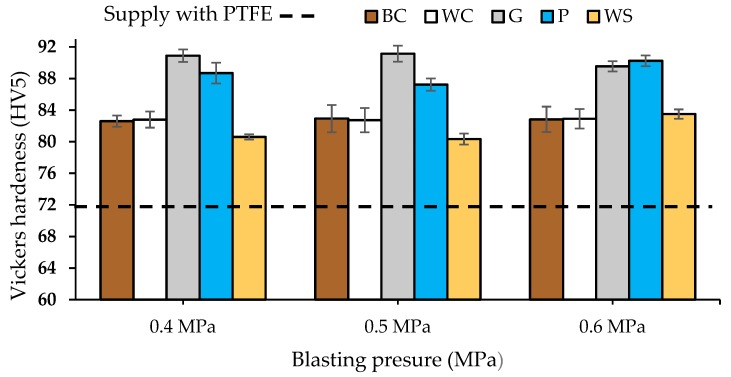
Vickers hardness values with loads of 5 kg (HV5) after stripping PTFE coatings on an EN AW 5182 substrate with different abrasives at 0.4, 0.5, and 0.6 MPa: (i) BC-brown corundum, (ii) WC-white corundum, (iii) G-glass microspheres, (iv) P-plastic particles, and (v) WS-walnut Shell.

The values of Vickers hardness with a load of 5 kp reached in the substrates after abrasive stripping were determined (Figure 5). The results were measured after projection with 0.4, 0.5, and 0.6 MPa. The application time was necessary for the effective removal of the coating.

**Figure 6:**
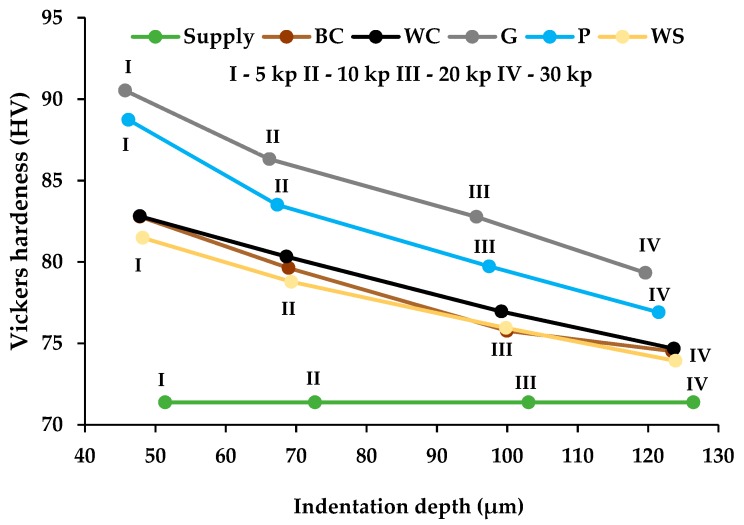
Vickers hardness with loads 5, 10, 20, and 30 kp versus penetration depth in ENAW5182 aluminium substrate, after sandblasting with various abrasives between 0.4 to 0.6 MPa for PTFE stripping.

The aluminium after the PTFE coating has 71.3 HV5 hardness. The graph in Figure 6 shows that, for all abrasives, after the removal of the coating, the hardness of the substrate increases.

The abrasives that caused the highest degree of hardening of the substrate were the glass microspheres followed by the plastic particles, with values between 88–91.5 HV5 against values of 82–83 HV5 in the case of corundum. These abrasives have lower hardness on the Mohs scale than white or brown corundum, 6 (glass) and 4 (plastic) vs. 9 (corundum), but the application time for the complete removal of PTFE is 2.5 to 3 times higher.

For walnut shell abrasiveness (2.5–3 Mohs scale), blasting times are 7 to 8 times greater than brown or white corundum. Despite the blasting time, the hardness remains between 80–83 HV5 and there is no noticeable increase. The lower hardness of the abrasiveness causes an increase in hardness, but this is limited and has little dependence on the abrasive application time.

On the other hand, it can be seen that each type of abrasive produces similar hardness levels for the blasting pressures tested 0.4, 0.5, and 0.6 MPa. For brown corundum, a hardness level between 82.6–82.9 HV5 has been obtained. For white conundrum a hardness level between 82.8–82.9 HV5 has been obtained while for glass microspheres values between 89.5–91.1 HV5 have been obtained. In the case of plastic particles, a hardness level between 87.2 and 90.2 HV5 has been obtained and for walnut shell values between 80.6 and 83.5 HV5 have been obtained. The increase in pressure has been compensated by the decrease in blasting time and vice versa and, therefore, the hardness remains nearly stable for the different blasting pressures.

The values, levels, and models of the increase in hardness and mechanical resistance that are achieved in the alloy studied are described in various models on projection with abrasives [39,40]. The results of the proposed work corroborate the increase.

In order to know the magnitude of the hardened thickness, the Vickers hardness versus depth has been studied. A Vickers indenter has been prepared with loads of 5, 10, 20, and 30 kp. The depth of the footprint on our sandblasted substrates has been measured for those specimens obtained with pressures ranging from 0.4 to 0.6 MPa. The results are shown in Figure 6.

It can be observed that the variation in hardness is practically linear and that the decrease of this property is more pronounced (greater slope) in those substrates sandblasted with glass microspheres and plastic particles than those with corundum and walnut shells. A law has been proposed that relates the Vickers hardness to the hardened thickness by means of a linear approximation (Table 4).

The laws proposed in Table 4 allow us to relate the Vickers hardness value to the depth hardened in the total thickness of the processed substrate. When studying the maximum hardening depth (p_max_) due to the impact of abrasives, it is enough to replace the HV value for the value of the hardness of the sample supplied with PTFE. Thus, the maximum hardened depth from greater to lesser is obtained by: (1) glass microspheres, (2) plastic particles, (3) white corundum, (4) brown corundum, and (6) walnut shell. The levels are ≈170 µm for glass microspheres and ≈150 µm for all other abrasives.

Under the criterion of obtaining high Vickers hardness values and thicknesses of substrates affected by high hardening, the best abrasives are the glass microspheres and plastic particles. Hardness increases have reached levels of 17–19 units of HV5. In the case of the two-step procedure, the substrate does not experience any improvement in hardness with respect to the state after the application of PTFE [8,32].

### 3.4. SEM Images

**Figure 7:**
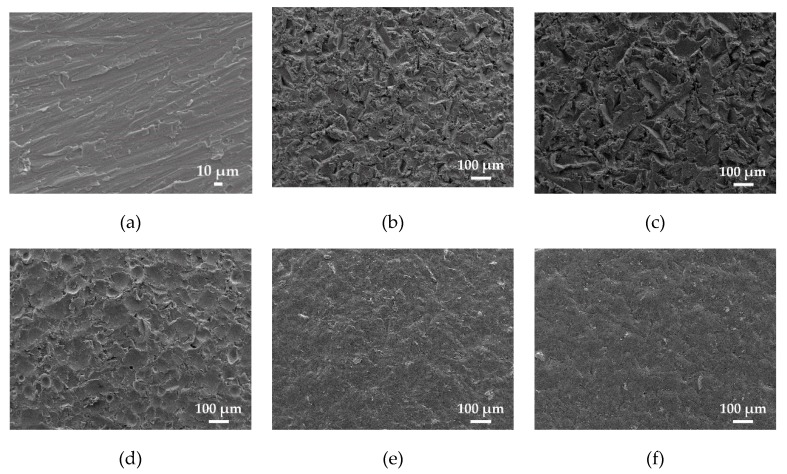
SEM images of the various sandblasted samples. (**a**) State of delivery, (**b**) brown corundum, (**c**) white corundum, (**d**) glass microspheres, (**e**) plastic particles, and (**f**) walnut shell.

Figure 7 shows the images obtained by the high-resolution scanning electron microscope in the state of supply and after stripping with each type of abrasive.

The most abrupt surfaces can be seen in Figure 7b,c, as anticipated by the result of roughness Ra ≥ 4–5 μm obtained using brown and white corundum. Cuttings and surface deformation of the substrate can be appreciated.

In the case of glass microspheres (Figure 7d), the value of Ra (3.5–4.5 µm) is relatively high and it is observed that there are traces corresponding to the spherical shape of the abrasive particles on the surface and, therefore, high values of Rz (35–37 µm) are obtained.

The plastic particles and walnut shells produce a low level of irregularities (Figure 7e,f) that are compatible with the values of Ra (2–3 µm) and Rz (15–20 µm) obtained.

For the abrasiveness of glass microspheres as well as for plastic particles, the removal of the coating did not take place by a cutting effect but by a compressive effect (the images do not show cuts). The compressive mechanism likely generates micro-fissures by compression and, consequently, eliminates the coating. This effect is similar to cold plastic deformation and the hardness of the substrate after stripping is the highest obtained in the experiments.

In the micrograph relating to the abrasive walnut shell, there are also no cuts on the substrate surface. Due to the smaller size and lower hardness, this abrasive produces a lower substrate hardening than glass microspheres or plastic particles, but similar to that produced by corundum abrasives.

The appearance of the SEM images is similar to those obtained in other works [41,42], for comparable abrasives used, and is compatible with the previously developed cutting models [40,43].

## 4. Conclusions

Considering the experimental study developed in this work, it can be indicated that, in order to strip PTFE coatings on Al-Mg substrates with abrasives in a single step, the following should occur.

- The highest stripping rates (0.6–0.7 cm^2^/s) were obtained with brown and white corundum abrasives. This occurs both at 0.4, 0.5, and 0.6 MPa. The rates obtained by this route (1 step) are lower than those obtained by conventional techniques (2 steps), even though the process is more respectful to the environment.
- The roughness levels Ra (2.5–4 µm) and Rz (30–35 µm) suitable for a correct surface preparation and for a later coating and reuse of the substrate were obtained with the abrasiveness of glass microspheres and with plastic particles in the pressure ranges tested (0.4, 0.5, and 0.6 MPa).
- The optimal values to increase the hardness of the substrate (17–19 pcs of HV5 with respect to the recrystallized state) and greater depth of affectation (170 µm) were produced with the abrasives constituted by glass microspheres and plastic particles for all the values of the projection pressures (0.4, 0.5, and 0.6 MPa). A law has been proposed that relates Vickers hardness to hardened thickness by linear approximation.
- All abrasives tested increase hardness on the substrate (10–19 units of HV5) in the case of a one-step procedure, unlike the process constituted in two steps. By contrast, stripping rates are significantly lower than 0.5–0.7 vs 1 to 1.2 cm^2^/s.

Therefore, in the one-step abrasive stripping process, the most suitable are glass microspheres and plastic particles. Although they have intermediate stripping rates, they are the ones that allow us to reach the adequate roughness during the cleaning process for a later coating with PTFE. In addition, they allow us to obtain high values of surface hardness, which is an essential property so that many pieces do not lose functionality after the thermal treatment applied for the PTFE sintering.

Lastly, it can be concluded that, although traditional procedures for removing PTFE coatings on Al-Mg substrates in two steps (carbonization + abrasive blasting) are faster than a single-step procedure (abrasive blasting), the latter improve the mechanical properties of the substrate and produce more suitable surface textures. Furthermore, the one-step procedure does not need to carbonize, does not emit combustion gases, and is environmentally more efficient.

## Figures and Tables

**Table 1:** Chemical composition (% by weight) of the EN AW-5182 alloy.

Si	Fe	Cu	Mn	Mg	Cr	Zn	Ti	Other	Al
0.10	0.20	–	0.40	4.08	0.15	–	0.05	–	Rest

**Table 2:** Application process, thickness, and colour of PTFE coating TFI-2531 N.

Products (Whitford)	Temperature (°C)/Time (min)	Thickness Layer 1 (µm)/Thickness Layer 2 + 3 (µm)	Colour
Layer 1: Quantum2 7131	120–150/5	13.5 ± 3.5/36.1 ± 3.2	Black
Layer 2 + 3: Quantum2 7232 + Eclipse 7353	410/15		

**Table 3:** Characteristics of the abrasives used in the study.

Abrasive	Mohs Hardness	Grain Size (µm)	Specific Weight (g/cm^3^)
Brown Corundum	9	600–425	3.94
White Corundum	9	425–300	3.98
Glass Microspheres	6	300–200	2.5
Plastic Particles	4	450–300	1.52
Walnut Shell	2.5–3	240–100	1.2–1.4

**Table 4:** Ratio of Vickers hardness (HV) to indentation depth p (µm) on EN AW-5182 substrates after blasting with various abrasives from 0.4 to 0.6 MPa for PTFE stripping.

Abrasive Types	Ratio between HV (Vickers Hardness) and p (Depth-µm)	p_max_ (µm)
Brown corundum (BC)	HV = −0.1110 × p + 87.167	146.2
White corundum (WC)	HV = −0.1074 × p + 87.807	152,9
Glass microspheres (G)	HV = −0.1469 × p + 96.755	172.7
Plastic particles (P)	HV = −0.1520 × p + 94.849	154.4
Walnut shell (WS)	HV = −0.0985 × p + 85.941	147.82

## References

[1] Holmberg K., Matthews A. (2009). Coatings Tribology: Properties, Mechanisms, Techniques and Applications in Surface Engineering.

[2] Gérard B. (2006). Application of thermal spraying in the automobile industry. Surf. Coatings Technol..

[3] Miller R.A. (1997). Thermal barrier coatings for aircraft engines: History and directions. J. Therm. Spray Technol..

[4] Cooper I., Tice P. (2001). Food contact coatings—European legislation and future predictions. Surf. Coatings Int. Part B Coatings Int..

[5] Dehghanghadikolaei A., Fotovvati B. (2019). Coating Techniques for Functional Enhancement of Metal Implants for Bone Replacement: A Review. Materials.

[6] Møller V.B., Dam-Johansen K., Frankær S.M., Kiil S. (2017). Acid-resistant organic coatings for the chemical industry: A review. J. Coatings Technol. Res..

[7] Ebnesajjad S., Khaladkar P.R. (2017). Fluoropolymer Applications in the Chemical Processing Industries: The Definitive User’s Guide and Handbook.

[8] Rodríguez-Alabanda Ó., Romero P., Guerrero-Vaca G. (2018). Evaluation of Substrates of Al-Mg and Aluminized Steel Coated With Non-Stick Fluoropolymers after the Removal of the Coating. Materials.

[9] Gardiner J. (2015). Fluoropolymers: Origin, Production, and Industrial and Commercial Applications. Aust. J. Chem..

[10] Thomas P. (1998). The use of fluoropdymers for non-stick cooking utensils. Surf. Coat. lnt..

[11] Teng H. (2012). Overview of the Development of the Fluoropolymer Industry. Appl. Sci..

[12] McKeen L.W. (2006). Fluorinated Coatings and Finishes Handbook: The Definitive User’s Guide.

[13] Rodríguez-Alabanda Ó., Romero P.E., Soriano C., Sevilla L., Guerrero-Vaca G. (2019). Study on the main influencing factors in the removal process of non-stick fluoropolymer coatings using Nd:YAG Laser. Polymers.

[14] Guerrero G.R., Sevilla L., Soriano C. (2015). Laser and pyrolysis removal of fluorinated ethylene propylene thin layers applied on en AW-5251 aluminium substrates. Appl. Surf. Sci..

[15] Drobny J.G. (2010). Technology of Fluoropolymers.

[16] Echt A., Dunn K.H., Mickelsen R.L. (2000). Automated Abrasive Blasting Equipment for Use on Steel Structures. Appl. Occup. Environ. Hyg..

[17] Yildizli K., Karamiş M.B., Nair F. (2006). Erosion mechanisms of nodular and gray cast irons at different impact angles. Wear.

[18] Childers S., Watsnr D.C., Stumpff P., Tirpak J.D. (1985). Evaluation of the Effects of A Plastic Bead Paint Removal Process on Properties of Aircraft Structural Materials. https://pdfs.semanticscholar.org/10a4/b844dbf083f91c0ffd3577ba83486217325c.pdf.

[19] Faíña A., Souto D., Deibe A., López-Peña F., Duro R.J., Fernández X. (2009). Development of a climbing robot for grit blasting operations in shipyards. Proc. IEEE Int. Conf. Robot. Autom..

[20] Carvalhão M., Dionísio A. (2015). Evaluation of mechanical soft-abrasive blasting and chemical cleaning methods on alkyd-paint graffiti made on calcareous stones. J. Cult. Herit..

[21] Guerrero G. (2013). Análisis comparativo de los procesos de eliminación de recubrimientos antiadherentes fluoropoliméricos en superficies metálicas entre tecnologías láser y pirolíticas. Ph.D. Thesis.

[22] Linke B.S. (2015). A review on properties of abrasive grits and grit selection. Int. J. Abras. Technol..

[23] Hu Z., Marshall C., Bicker R., Taylor P. (2007). Automatic surface roughing with 3D machine vision and cooperative robot control. Rob. Auton. Syst..

[24] Souto D., Faiña A., Deibe A., Lopez-Peña F., Duro R.J. (2012). A robot for the unsupervised grit-blasting of ship hulls. Int. J. Adv. Robot. Syst..

[25] Dechezleprêtre A., Sato M. (2017). The impacts of environmental regulations on competitiveness. Rev. Environ. Econ. Policy.

[26] Flynn M.R., Susi P. (2004). A review of engineering control technology for exposures generated during abrasive blasting operations. J. Occup. Environ. Hyg..

[27] King R.G. (2014). Surface Treatment & Finishing of Aluminium.

[28] Karbouj R., Desloges I., Nortier P. (2009). A simple pre-treatment of aluminium cookware to minimize aluminium transfer to food. Food Chem. Toxicol..

[29] Materiales Metálicos (2006). Ensayo de Dureza Vickers. Parte 1: Método de ensayo.

[30] Reitz W.E. (1994). Coating-removal techniques: Advantages and disadvantages. JOM.

[31] Geng S., Sun J., Guo L. (2015). Effect of sandblasting and subsequent acid pickling and passivation on the microstructure and corrosion behavior of 316L stainless steel. Mater. Des..

[32] Li D., Liu B., Man Y., Xu K. (2001). Effects of a Modified Sandblasting Surface Treatment on Topographic and Chemical Properties of Titanium Surface. Implant Dent..

[33] Guerrero G.R., Sevilla L., Soriano C. (2014). Ablación láser de recubrimientos de politetrafluoretileno (PTFE) aplicados sobre sustratos EN AW-5251. Rev. Metal..

[34] Draganovská D., Ižaríková G., Guzanová A., Brezinová J. (2018). General Regression Model for Predicting Surface Topography after Abrasive Blasting. Metals.

[35] Fernández-Abia A.I., Barreiro J., López de Lacalle L.N., González-Madruga D. (2014). Effect of mechanical pre-treatments in the behaviour of nanostructured PVD-coated tools in turning. Int. J. Adv. Manuf. Technol..

[36] Lewiński J., Niżankowski C. (2017). The Influence of the Parameters of the Thin Walled Metal Sheets Shot Peening on the Technological Quality of Work Surfaces. https://stc.fs.cvut.cz/history/2017/sbornik/papers/pdf/6616.pdf?_=1492168543.

[37] Wang S., Li Y., Yao M., Wang R. (1998). Compressive residual stress introduced by shot peening. J. Mater. Process. Technol..

[38] Curtis S., De Los Rios E.R., Rodopoulos C.A., Levers A. (2002). Analysis of the effects of controlled shot peening on fatigue damage of high strength aluminium alloys. Int. J. Fatigue.

[39] Jackson M.J., Davim J.P., Jackson M.J., Davim J.P. (2011). Machining with Abrasives.

[40] Haldar B., Adak D.K., Ghosh D., Karmakar A., Habtamu E., Ahmed M., Das S. (2018). Present status and some critical issues of abrasive jet materials processing: A review. Procedia Manuf..

[41] Calignano F., Manfredi D., Ambrosio E.P., Iuliano L., Fino P. (2013). Influence of process parameters on surface roughness of aluminum parts produced by DMLS. Int. J. Adv. Manuf. Technol..

[42] Veloz N.F. (1993). Practical Aspects of Using Walnut Shells for Cleaning Outdoor Sculpture. APT Bull. J. Preserv. Technol..

[43] Hashimoto F., Yamaguchi H., Krajnik P., Wegener K., Chaudhari R., Hoffmeister H.-W., Kuster F. (2016). Abrasive fine-finishing technology. CIRP Ann..

